# Effects of Exercise in Patients with Obstructive Sleep Apnoea

**DOI:** 10.3390/clockssleep3010013

**Published:** 2021-03-03

**Authors:** Rodrigo Torres-Castro, Luis Vasconcello-Castillo, Homero Puppo, Ignacio Cabrera-Aguilera, Matías Otto-Yáñez, Javiera Rosales-Fuentes, Jordi Vilaró

**Affiliations:** 1 Department of Physical Therapy, Faculty of Medicine, University of Chile, Santiago 8380453, Chile; l.vasconcello.c@gmail.com (L.V.-C.); homeropuppo@gmail.com (H.P.); jrosalesfuentes@gmail.com (J.R.-F.); 2 Department of Kinesiology, University of Talca, Talca 3460000, Chile; ignaciocabrera.a@gmail.com; 3 Kinesiology School, Universidad Autónoma de Chile, Santiago 7500912, Chile; matiasottokine@gmail.com; 4 Grupo de Investigación Global Research on Wellbeing (GRoW), Facultad de Ciencias de la Salud Blanquerna, Universitat Ramón Llull, 08025 Barcelona, Spain; jordi.gestos@gmail.com

**Keywords:** sleep apnoea, exercise, physical exercise, oropharyngeal exercises, respiratory muscle training

## Abstract

Obstructive sleep apnoea (OSA) constitutes a public health problem, with various systemic consequences that can increase cardiovascular morbidity and mortality as well as increase healthcare expenditure. This review discusses the rationale and effects of using general physical exercise, oropharyngeal exercises, and respiratory muscle training as an adjunctive treatment for patients with sleep apnoea. The recommended treatment for OSA is the use of continuous positive airway pressure, which is a therapy that prevents apnoea events by keeping the airways open. In the last decade, coadjuvant treatments that aim to support weight loss (including diet and physical exercise) and oropharyngeal exercises have been proposed to lower the apnoea/hypopnoea index among patients with OSA. Based on the available evidence, health professionals could decide to incorporate these therapeutic strategies to manage patients with sleep apnoea.

## 1. Introduction

Obstructive sleep apnoea (OSA) is generated by the intermittent collapse of the upper airway during sleep, which leads to transient asphyxia [1]. This condition affects 9% to 38% of the general adult population (aged over 18 years) constituting a public health concern, particularly in overweight and obese subjects [2]. OSA is associated with a wide range of important health consequences, including daytime sleepiness, fatigue, cognitive impairment, and metabolic and cardiovascular diseases [3]. Polysomnography (PSG) is the gold standard method for diagnosing OSA. PSG provides an apnoea/hypopnoea index (AHI), which is the most common and widely used outcome to determine the severity of OSA [4]. The AHI is the sum of all events (apnoeas and hypopnoeas) divided per hour of sleep [5]. An AHI of 5 to 14 is defined as mild, 15 to 29 is defined as moderate, and over 30 is defined as severe [6].

The recommended treatment for OSA is the use of continuous positive airway pressure (CPAP) [3,7,8], which a therapy that prevents apnoea events by keeping the airways open. Treatment with CPAP is cost-effective [9], decreases morbidity and mortality in cardiovascular diseases [10,11], and reduces the risk of drowsiness-related traffic accidents [12]. However, low adherence to CPAP reduces the benefits obtained by patients receiving this therapy. It is estimated that between 29% and 83% of patients receiving CPAP are non-adherent with therapy (<4 h) [13]. The usage of CPAP is low among patients with severe sleep apnoea but little sleepiness [14]. Adherence is essential to decrease cardiovascular morbidity and, consequently, the associated economic cost. OSA has a substantial economic impact, resulting from the occurrence of cardiovascular events and metabolic disease. The literature has consistently shown that patients with OSA have higher hospital admission rates and usage of medical services [15,16].

Untreated OSA can have several significant consequences, including hypoxemia and hypercapnia, fragmented sleep, heart rhythm fluctuations, and blood pressure increase [17]. These effects can lead to long-term sequelae, such as cardiovascular comorbidities [17], cognitive deterioration [18], and premature death [19].

Obesity is the most critical risk factor for developing OSA, being present in 70% of the patients [20]. Indeed, the prevalence of obesity has been increasing worldwide, and OSA in this population is rising at an alarming rate [21]. Additionally, the literature has shown that small weight reductions have reduced AHI [22].

In the last decade, coadjuvant treatments that aim to support weight loss (including diet [23], physical exercise [24,25]), and oropharyngeal exercises [26,27] have been proposed to lower the AHI among patients with OSA. Indeed, regular physical activity is associated with decreased OSA and improved sleep efficiency [22,28]. Despite the potential benefits of weight-loss interventions through exercise, patients with OSA rarely adhere to rehabilitation programmes, which commonly require scheduled hospital visits or the use of sophisticated training equipment (e.g., treadmills, cycle ergometers) that are not always available to the patient outside of the rehabilitation programme [29,30,31].

## 2. Methods

The aim of this literature review was to discuss the rationale and effects of using general physical exercise, oropharyngeal exercises, and respiratory muscle training as an adjunctive treatment for patients with sleep apnoea. The question that guided the review process was: What are the effects of physical exercise, oropharyngeal exercises, and respiratory muscle training in patients with obstructive sleep apnoea? A bibliographic search was conducted to identify relevant studies. We used Medline/PubMed and EMBASE from 1990 until September 2020. The following keywords were used: “exercise” or “physical training” or “aerobic training” or “oropharyngeal exercise” or “myofunctional therapy” or “respiratory muscle training” or “inspiratory muscle training” and “obstructive sleep apnoea”. The included studies in the review were (1) studies in humans, (2) studies with a duration of at least 1 month, and (3) studies in which the main outcome was apnoea–hypopnoea index. The studies finally selected were incorporated into the text by mutual agreement between the authors.

## 3. Effects of Physical Exercise

Physical exercise has been suggested to lower the AHI in OSA patients [24,32]. Indeed, physical exercise programmes are associated with decreased OSA prevalence, lower AHI scores, improved sleep efficiency, and less daytime sleepiness [22,33]. While the mechanisms underlying these beneficial effects in OSA patients are not totally understood, it is known that exercise can reduce body mass and fat mass, which have been related to significant reductions in the AHI [34]. The possible mechanisms are related to the regulation of hormonal activity within adipose tissue and other major endocrine organs [35], the reduction of leptin levels and enhanced leptin sensitivity [36], the circulation of adiponectin [37], the increase of growth hormone secretion that is related to the reduction of abdominal fat in adults [38], the potent anti-inflammatory properties of exercise [39], the improvement of insulin sensitivity [40], and the reduction in triglycerides and total cholesterol [41]. However, several studies have found that the beneficial effects of exercise on OSA are not only related to reduced body mass [31,33,42], it could also be related to other factors such as body composition, especially in regard to the reduction in fat mass [43].

Desplan et al. studied adults with moderate–severe OSA aged from 35 to 70 years and performed a 4-week inpatient rehabilitation programme, including 24 supervised exercise sessions (15 min of warming up muscles, 45 min of cycle ergometer endurance training, 30 min of muscles reinforcement with resistance training, 15 min of stretching, and 15 min of postural and balance exercises) versus a control group [24]. Compared to controls, participants randomised to the intervention group decreased in AHI (40.6 ± 19.4 vs. 28.0 ± 19.3; *p* < 0.001), oxygen desaturation index (ODI), and arousal index, which occurred in conjunction with a significant decrease in body mass index (BMI), neck circumference, fat mass, fasting glucose, and diastolic blood pressure [24]. Additionally, the intervention group improved sleepiness, anxiety, depression, and quality of life. Kline et al. studied adults aged from 18 to 55 years with BMI ≥ 25 kg/m^2^ and moderate-severe untreated OSA who performed moderate-intensity exercise (a gradual increase from 50 min/week to 150 min/week) plus resistance training for 12 weeks [25]. Compared with the control group, exercise resulted in a significant AHI reduction (exercise: 32.2 ± 5.6 to 24.6 ± 4.4, control group: 24.4 ± 5.6 to 28.9 ± 6.4; *p* < 0.01, medium effect size [Hedges’ d −0.44]) as well as significant changes in ODI (*p* = 0.03). In addition, significant improvements were achieved in depressive symptoms, fatigue and vigour, sleepiness, functional impairment, and aspects of QOL. Reductions in AHI and ODI were achieved without a significant body mass decrease.

**Figure 1:**
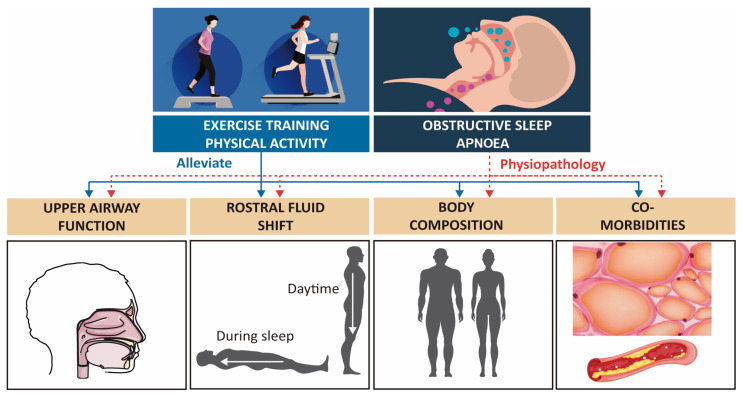
Hypothetical relationship between physical exercise and sleep apnoea. In obstructive sleep apnoea (OSA) patients, the strength and fatigability of the upper airway dilators have been shown to be altered; the rostral fluid shift contributes to their pathogenesis; the elevated body mass index (BMI) is a significant risk factor; and the OSA is often accompanied by cardiovascular and metabolic comorbidities. Physical activity and exercise training has been shown to alleviate OSA. Based on Mendelson et al. [33].

Weight loss has not been related to significant AHI reductions, and it is likely that the improvements are due to changes in body composition (especially reduction in fat mass) [33,34]. Several mechanisms may explain these improvements. In the case of exercise, physical exercise could reduce the AHI by reducing fat deposition in the anatomical structures surrounding the airway and tongue [44]. Additionally, exercise may cause significant abdominal adiposity reductions, independently of weight loss [45] (Figure 1). This fact is important, because abdominal visceral fat accumulation impairs diaphragmatic excursion, and chest wall obesity impairs rib cage expansion [29]. This mechanism is mediated by the liberation of catecholamines [46], which have greater lipolytic action in visceral fat than they do in subcutaneous fat [47].

It has been shown that fluid that accumulated in the legs during the day (due to gravity and the diminution of the muscular pump activity when lying down at night) is redistributed rostrally [48]. This rostral redistribution during the night, especially towards the neck and thorax, may play a role in the pathogenesis of OSA [49]. The redistribution of fluid in the neck can increase pressure on the tissue surrounding the upper airway, reducing its size and increasing its collapsibility, which is a predisposition towards OSA [48]. Interventions that reduce fluid accumulation in the legs, such as diuretics and wearing compression stockings, can attenuate OSA [50,51]. On the other hand, the increase of sedentary patients’ physical activity level with OSA has been associated with a reduction in AHI, independent of body mass. This situation occurs concomitantly with a reduction in fluid shifting from the legs to the pharynx during the night [52] (Figure 1).

The mechanisms that explain the improvement in AHI without BMI change following exercise training have not been elucidated. The potential mechanisms are (1) rostral fluid shift, (2) increased strength and fatigue resistance of the upper airway dilators, decreased nasal resistance, and increased respiratory stability through deeper sleep, (3) body composition modification and changes in fat mass distribution, and (4) changes in sleep efficiency [33].

## 4. Effects of Oropharyngeal Exercises

Oropharyngeal exercises (myofunctional therapy) consists of the tongue, soft palate, facial, and functional exercises, which aim to improve the muscles that keep the upper airway open [53]. Specifically, the genioglossus and pharyngeal musculatures play an essential role in OSA and can be trained through oropharyngeal exercises [54]. Recent scientific evidence supports oropharyngeal exercises commonly used in speech and dysphagia rehabilitation for treating OSA [54]. Possible benefits of these exercises include a reduction in neck circumference, snoring, subjective sleepiness, and AHI, as well as improved quality of life [27,54]

**Figure 2:**
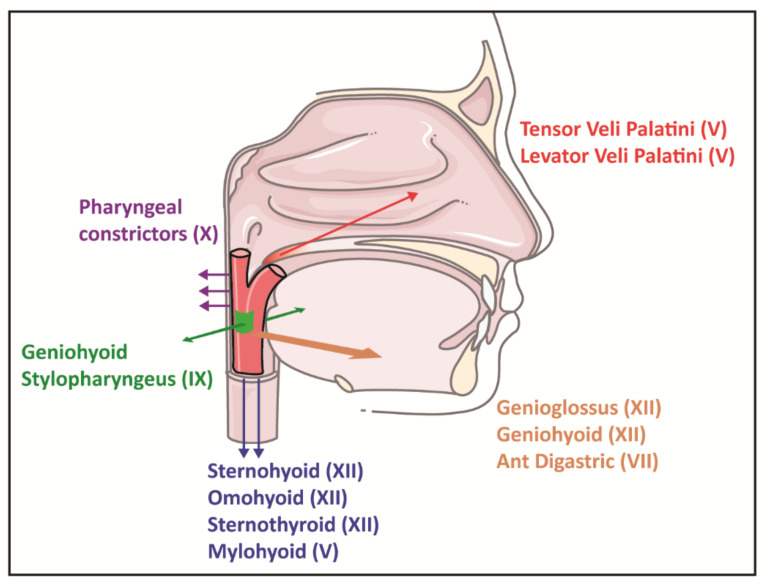
Representation of the upper airway in obstructive sleep apnoea: a reliance on upper airway dilator muscles for patency [55]. The airway is narrowed but remains patent in wakefulness in large part because of crucial dilator muscles, which is labelled in the diagram with cranial nerve innervations between parentheses. The overall force vectors are indicated with arrows and are shown on both the diagram and images. Upward-directed arrows (red colour) signify force vectors for tensor veli palatini and levator palatini muscles in raising the soft palate (uvula) and lateral walls. Since the pharynx is collapsible at all tangents, multiple muscle groups must act in concert to prevent the pharynx from collapsing.

There are multiple muscle groups involved in maintaining upper airway patency in persons anatomically predisposed to obstructive sleep-disordered breathing [55]. The oropharynx is highly collapsible from multiple directions; most individuals with a predisposition to the sleep-related collapse of the upper airway rely on opposing muscle groups to work in unison to prevent upper airway collapse (Figure 2). The genioglossus is the largest and most potent upper airway dilator; however, their muscle activation alone may be insufficient to reduce pharyngeal collapsibility [55].

Diaféria et al. conducted a clinical trial in which 100 male patients (25–65 years) were divided into four groups: oropharyngeal exercises group, placebo group, CPAP group, and a combination of CPAP and oropharyngeal exercise group [56]. The experimental group and the combination group underwent a 3-month intervention of 20 min daily of oropharyngeal exercises (including the soft palate, tongue, and facial muscle exercises as well as stomatognathic function exercises). It was shown that oropharyngeal exercises reduce daytime sleepiness measured by the Epworth sleepiness scale and the severity of apnoeas by decreasing the AHI; the authors did not find greater benefits if associated with CPAP; however, they reported better adherence to the device during the first week of treatment [56].

Guimaraes et al. recruited 31 patients with moderate OSA; the sample mainly consisted of overweight and/or obese middle-aged men (mean age close to 50 years), they were randomised to 3 months daily (30 min consisting of exercises involving the tongue, soft palate, and lateral pharyngeal wall) sham therapy (n = 15), or a set of oropharyngeal exercises (n = 16) [26]. The intervention group had a significant decrease in neck circumference, snoring frequency, snoring intensity, daytime sleepiness, sleep quality, and OSA severity (AHI) [26]. Several studies show that the primary outcomes that improve with the oropharyngeal exercises are daytime sleepiness and sleep quality [57,58].

The different training protocols for these muscles are focused on improving endurance, neuromuscular coordination, stability, and increasing the tone of this group of muscles [56]. Muscle tone plays a vital role in the appearance of sleep apnoeas, mainly in obese and overweight patients who have greater fat deposits on the tongue and neck [44]. Increasing load in the upper airway results in a tendency to collapse, especially if the oropharyngeal muscles have a decreased tone; this is exacerbated at night when there is a decrease in muscle tone, favouring the appearance of sleep apnoeas [59].

## 5. Effects of Respiratory Muscles Training

Respiratory muscle training (RMT) aims to strengthen the diaphragm, external intercostals, and accessory respiratory muscles; this training is performed by breathing against a specific resistance through an adjustable valve [60]. In addition, RMT can increase the tone and neuromuscular control of the pharyngeal muscles regardless of the increase in muscle mass or strength. Increased tone of the pharyngeal dilator muscles provided by RMT may reduce the upper airway’s tendency to collapse during sleep.

Erturk et al., in a clinical trial, used a protocol of inspiratory muscle training (IMT) of 15 min twice a day, seven days per week for 12 weeks using a Threshold IMT device (Respironics, USA) from 30% of the maximal inspiratory pressure (MIP) in clinically stable OSA patients not receiving CPAP therapy [61]. This study found significant decreases in snoring severity and frequency, fatigue, and sleep quality. The IMT group also increases MIP and maximal expiratory pressure (MEP) compared with the control group. Nevertheless, there were no significant changes in the severity of apnoeas (AHI). Other studies have reported an increase in MIP after a training protocol [62,63], despite high loads (50–75% of MIP), and they also do not report improvements in AHI. Although the authors suggest that IMT would increase pharyngeal strength and control, the lack of a significant reduction in AHI suggests that IMT may affect the diaphragm and respiratory accessory muscles, confirming the findings that there is no association between respiratory muscle strength and AHI in OSA.

On the other hand, expiratory musculature training has been shown to improve apnoea severity. Kuo et al. reported significant amelioration in sleep apnoeas, sleep quality, and MEP in nine patients with moderate and mild OSA, who were subjected to a 5-week expiratory muscle training protocol with a load of 75% of MEP: four patients with a frequency of 5 days/week and five patients with a frequency of 3 days/week [64]. This finding may be related to the neuromuscular control of the pharynx’s intrinsic musculature. Wheeler et al., in 20 healthy subjects between 18 and 35 years, reported that neuromuscular activation patterns in the submental muscle were longer in the group undergoing expiratory training (25 and 75% of MEP) [65].

Although IMT has not been shown to improve the severity of apnoea, it has shown improvements in other outcomes that may be important for patients with OSA, such as sleep quality and fatigue. If we add expiratory muscle training, we can achieve an intervention that improves several of the problems that sleep apnoeas generate in patients, mainly in those who do not tolerate CPAP therapy. Finally, an important point to consider in future studies is to evaluate if there is any different effect in patients who, in addition to having OSA, have congestive heart failure, COPD, or some other morbidity.

## 6. Conclusions

OSA constitutes a public health problem, with various systemic consequences that can increase cardiovascular morbidity and mortality and economic expenditure on health. That is why adjunctive treatments such as physical exercise or oropharyngeal exercises can improve the AHI and quality of life in patients with OSA, not only in those who use CPAP but also in the group with low adherence to therapy. However, it is essential to consider that the respiratory, oropharyngeal, or general muscles training must be followed with physiological principles of overload, specificity, and variability to achieve favourable results [60]. Nevertheless, in oropharyngeal exercises and respiratory muscle training, the frequency of training and duration continue to be a matter of discussion. The key to prescribing training is the correct dosage of its intensity or load, as well as the total time or volume of training [60].

Physical exercise is a well-known therapeutic strategy for weight loss, which is very important to consider since obesity is among the most critical causes of OSA. Specific training of the respiratory muscles, both inspiratory and expiratory, appears to be a promising strategy to be better explored in future studies. Health professionals who work with OSA patients should be aware of the benefits of exercise so that they can incorporate them into their therapeutic strategies with this population.

## Data Availability

Not applicable.
